# Climate Filtering Governs the Distribution of Invasive Insect Assemblages Within Biodiversity Conservation Priority Areas in Guangxi, China

**DOI:** 10.3390/insects17050524

**Published:** 2026-05-20

**Authors:** Xin Huang, Dan Xiang, Zhi-Gang Yang, Xue-Kui Huang, Xiang-Lin Li, Jin-Long Huang, Rui-Gang Yang

**Affiliations:** 1 Guangxi Research Academy of Environmental Sciences, Nanning 530022, China; huangxin@gxhky.cn (X.H.); xiangdan@gxhky.cn (D.X.); crazbou@163.com (Z.-G.Y.); huangxk10@163.com (X.-K.H.); lixianglin@gxhky.cn (X.-L.L.); 2 Key Laboratory of Ecology of Rare and Endangered Species and Environmental Protection (Guangxi Normal University), Ministry of Education, Guilin 541006, China; jl_huang@163.com

**Keywords:** invasive alien insects, climate filtering, land cover, biodiversity conservation priority area

## Abstract

Biological invasions are a primary driver of biodiversity loss, with significant socio-economic repercussions. Therefore, understanding the factors influencing the assembly of invasive insect assemblages in Biodiversity Conservation Priority Areas is essential for formulating effective management strategies. This study examined the invasive alien insect fauna across three such regions in Guangxi, China, from April to November 2022, employing a systematic grid-based sampling design that encompassed 84 grids. A total of nineteen invasive alien insects from seven orders were identified, with Coleoptera (26.3%), Hymenoptera (21.1%), and Diptera (15.8%) being the most prevalent. *Bactrocera cucurbitae* Bezzi, 1913 (57.1% occurrence frequency) and *Blattella germanica* Linnaeus, 1767 (53.6%) were the most frequently observed species. Species richness exhibited significant variation among the three areas, peaking in the Western Guangxi and Southern Guizhou Limestone Area. The assemblages’ composition displayed high beta diversity, as indicated by a total Sørensen dissimilarity of 0.645, primarily driven by species turnover (0.474) rather than nestedness (0.171), which suggests the presence of distinct species assemblages across the areas. Generalized linear mixed models and Canonical Correspondence Analysis identified precipitation and temperature as the primary factors influencing species richness and assemblages’ composition, respectively. Hierarchical partitioning indicated that climatic variables, specifically precipitation (48.0%) and temperature (32.0%), collectively accounted for the majority of the variation in species richness, significantly surpassing the impact of local land cover (20.0%). In contrast, contemporary anthropogenic land use factors exhibited no significant effect. Our findings illustrate that while human activities serve as the ultimate drivers of species introductions, the subsequent assembly of invasive insect assemblages within these Biodiversity Conservation Priority Areas are predominantly shaped by climatic conditions. This highlights the essential role of climate suitability in determining the success of post-introduction establishment. Therefore, we recommend the incorporation of high-resolution climate-matching models into biosecurity screening and monitoring frameworks for Biodiversity Conservation Priority Areas, with targeted surveillance efforts directed toward regions exhibiting the highest climatic suitability.

## Simple Summary

Biological invasions pose a significant threat to global biodiversity. Understanding the drivers that enable invasive alien insects to establish in new regions is therefore vital for effective conservation. This study aimed to identify the influencing factors of the distribution of invasive alien insects. We used Canonical Correspondence Analysis and generalized linear mixed models to analyze influencing factors, such as climate and land cover. Our key finding is that local climate was the primary factor determining the distribution of these invasive insects. In contrast, contemporary human land use had little effect at this regional scale. This indicates that while human activity facilitates species introductions, the subsequent establishment and assemblages are predominantly filtered by climatic conditions. We recommend using climate-matching data to identify areas of high invasion risk within protected regions and to focus monitoring and prevention efforts accordingly.

## 1. Introduction

Invasive alien insects (IAI) are the non-native insect species that have established populations outside their natural distribution range and, through dispersal, exert negative impacts on the ecosystems, native species, or human well-being of the invaded areas [1]. IAI represent a significant and growing threat to global ecosystems, agriculture, and human health. Their remarkable adaptability and high reproductive rates contribute to considerable crop losses, forest degradation, and the disruption of essential ecosystem functions and services. Quantitatively, invasive terrestrial invertebrates have incurred an estimated cost of US$ 712.44 billion to the global economy up to 2020, with an astonishing 88% of these costs attributed solely to IAI [2,3]. These invasive species drive biodiversity loss and species extinctions through mechanisms such as resource competition, disease transmission, and predation. For example, the invasive ant *Solenopsis invicta* Buren, 1972 can disrupt mutualistic networks by replacing native ants in their interactions with honeydew-producing Hemiptera, potentially leading to the loss of critical food resources for native ants and altering the dynamics of Hemiptera populations and their natural enemies [4]. Females of *Stictocephala bisonia* Kopp & Yonke, 1977 create deep incisions in the bark for oviposition, leading to the entry of wood-destroying fungi and pathogens, which disrupt sap flow and nutrient supply, resulting in branch dieback and bark necrosis; in severe cases, young plants cease growth and die [5]. In agricultural systems, pests such as the *Spodoptera frugiperda* J.E.Smith, 1797 and fruit flies (Tephritidae) can inflict direct yield losses exceeding 30% due to feeding damage [6,7]. Additionally, IAI serve as vectors for human diseases, as illustrated by *Aedis aegypti* (Linnaeus, 1762)’s role in transmitting dengue fever [8]. Despite their disproportionate economic and ecological impacts, IAI have historically garnered less research attention compared to other taxonomic groups [9].

The rapid spread and establishment of IAI have been driven by the interplay of climate change and anthropogenic disturbances. As temperatures rise and precipitation patterns change, non-native insect species may encounter increasingly favorable conditions encouraging their establishment and spread [10]. Rising temperatures can shorten insect development cycles and expand climatically suitable ranges, as evidenced by studies on *Diaphorina citri* Kuwayama, 1908 [11,12]. Altered precipitation patterns modify habitats; for example, *S. invicta* requires at least 510 mm of annual precipitation to thrive, which limits its presence in arid regions to irrigated areas, whereas excessive rainfall can devastate ant colonies [13]. Simultaneously, agricultural expansion and urbanization disrupt landscape connectivity: cropland fragmentation can diminish the efficacy of natural enemies, while artificial surfaces such as road networks create corridors for dispersal [14,15]. Climate change, by generating unoccupied climatic niches, synergistically interacts with human-mediated dispersal pathways (e.g., road networks) to connect and colonize these new habitats, thereby significantly facilitating the establishment and spread of IAI and heightening their ecological risks [16,17].

Biodiversity hotspots and conservation priority areas, which are characterized by high endemism and ecological sensitivity, are at an increased risk of invasion due to global environmental change [18]. While substantial research has concentrated on propagule pressure during the introduction stage of IAI, particularly as influenced by international trade, significantly less attention has been directed toward the critical environmental filters that operate during the post-introduction establishment stage, especially at regional scales and within biodiversity hotspots [19,20,21]. This study seeks to address this gap by examining three terrestrial Biodiversity Conservation Priority Areas (BCPAs) in Guangxi, China: the Western Guangxi and Southern Guizhou Limestone Area (WGSGL-BCPA), the Nanling Mountains Area (NLM-BCPA), and the Southwestern Guangxi Mountain Area (SWGM-BCPA). An invasive insect assemblage is a grouping of invasive alien insect species that co-occur in the same place and at the same time [22]. Utilizing a systematic grid-based sampling design and multi-dimensional diversity analyses, we aim to (1) characterize the α-diversity (species richness) and β-diversity (turnover and nestedness components) of invasive insect assemblages in three terrestrial Biodiversity Conservation Priority Areas (BCPAs) of Guangxi, China—specifically, the Western Guangxi and Southern Guizhou Limestone Area (WGSGL-BCPA), the Nanling Mountains Area (NLM-BCPA), and the Southwestern Guangxi Mountain Area (SWGM-BCPA), and (2) quantify the relative contributions of climatic factors (e.g., temperature; precipitation) versus land cover variables (e.g., cropland; forest) in driving the observed diversity patterns. Our findings will provide a scientific benchmark for monitoring prioritization and will inform climate-adaptive management strategies within these ecologically vulnerable regions.

## 2. Materials and Methods

### 2.1. Study Area and Systematic Sampling Design

The study area consisted of three inland terrestrial Biodiversity Conservation Priority Areas (BCPAs) in Guangxi, China, as defined by China’s Biodiversity Conservation Strategy and Action Plan (2011–2030). These BCPAs, each exhibiting unique biogeographic and ecological gradients, were selected to systematically evaluate the spatial patterns of IAI risks: the WGSGL-BCPA, characterized by karst topography (approximately 15,021 km^2^); the NLM-BCPA, a vital ecological transition zone (approximately 27,808 km^2^); and the SWGM-BCPA, which features a tropical marginal climate (approximately 28,116 km^2^). Based on biodiversity status, resource endowment, and socio-economic conditions, each BCPA was further categorized into three functional zones: Zone I, comprising legally established protected areas such as nature reserves and scenic spots; Zone II, encompassing areas with rich biodiversity, fragile ecosystems, or significant ecological functions that lack statutory protection; and Zone III, consisting of urban built-up areas, development zones, and intensively managed agricultural, pastoral, or fishery areas subject to substantial anthropogenic disturbance. The areal proportions of these zones within each BCPA were as follows: WGSGL-BCPA—Zone I: 30.9%, Zone II: 57.7%, and Zone III: 11.4%; NLM-BCPA—Zone I: 29.5%, Zone II: 62.9%, and Zone III: 7.6%; and SWGM-BCPA—Zone I: 32.0%, Zone II: 48.4%, and Zone III: 19.6%.

**Figure 1:**
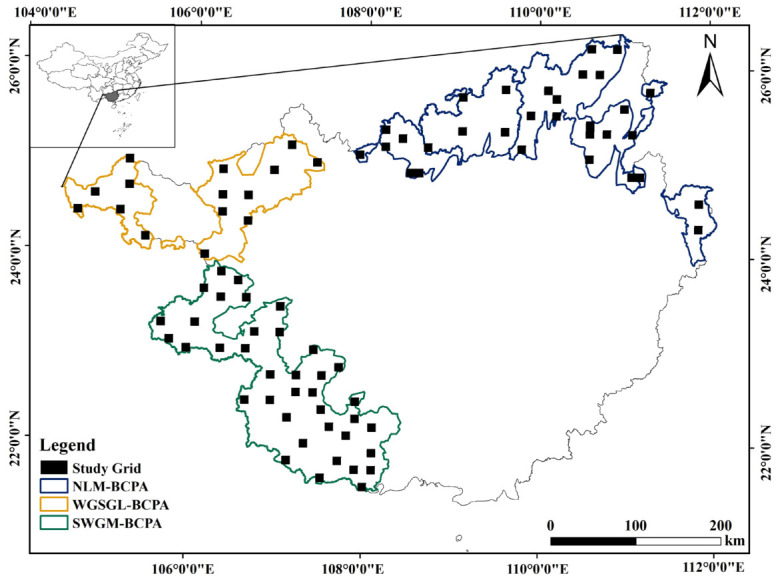
Location of the study area (three Biodiversity Conservation Priority Areas) and sampling grid.

A systematic spatial sampling framework was established by overlaying the entire study area with a 10 km × 10 km grid, resulting in approximately 830 candidate grid cells. To ensure a minimum sampling intensity of 10%, a total of 84 grid cells were selected as survey sites (Figure 1). The distribution of sites among the three BCPAs was determined using a stratified systematic sampling approach, with allocations of 15 for WGSGL-BCPA, 31 for NLM-BCPA, and 38 for SWGM-BCPA, weighted by two key dimensions. Initially, a baseline distribution was computed based on the relative area of each BCPA, resulting in an approximate ratio of 21:39:40%. Subsequently, this baseline was adjusted in accordance with the internal functional zoning, designating Zone III (areas of high human activity) as a potential high-risk zone for species introduction and establishment. As a result, survey efforts were deliberately directed towards BCPAs with a higher proportion of Zone III. The SWGM-BCPA, exhibiting the highest Zone III proportion (19.6%) and strategically positioned at the border to facilitate species introduction, was allocated an expanded quota (38 sites) exceeding its area-based allotment. Conversely, the WGSGL-BCPA, with the lowest Zone III proportion (11.4%) and lower anticipated invasion pressure, received a diminished allocation (15 sites). The NLM-BCPA, the most extensive in area and predominantly characterized by Zone II (62.9%) signifying a crucial conservation-activity interface, necessitated an ample number of sites to encompass its intricate ecological gradients; hence, its final allocation (31 sites) closely corresponded to the area-based approximation.

### 2.2. Field Survey and Data Collection

Field surveys were conducted during the primary insect activity season from April to November 2022. To thoroughly sample both diurnal and nocturnal IAI taxa, an integrated approach combining transect walks and light trapping was employed. Within each pre-selected 10 km × 10 km survey grid, four transects were established, with each transect measuring at least 0.5 km in length. The design of these transects adhered to two fundamental principles: (1) a systematic layout to ensure comprehensive coverage of typical ecosystems while minimizing spatial overlap; (2) practical accessibility, emphasizing the use of existing pathways and focusing on natural protected areas, border ports, adjacent routes, and regions with a documented history or high potential for invasive species occurrence. Diurnal surveys were performed utilizing the pollard walk technique, with survey personnel walking steadily along predetermined transects. Airborne insects were captured using standard insect sweep nets (38 cm mouth diameter; 70 cm depth) employing rhythmic figure-eight sweeping motions. To collect insects residing in foliage, branches, or fruits, the beat sheet method was utilized. This involved placing a white enamel tray or umbrella-shaped collector beneath the vegetation, which was then shaken or tapped to dislodge specimens onto the collection surface. Nocturnal surveys were conducted on clear, calm nights between 21:00 and 02:00 using standardized light traps. Any collected specimens suspected to be invasive species were promptly preserved in vials containing absolute ethanol and transported to the laboratory for identification. Species identification primarily relied on consulting the taxonomic keys and descriptions provided in Invasive Alien Species in China (Revised Edition) [23]. Subsequently, confirmed records of invasive insect species were compiled into a site-by-species presence–absence matrix, forming the foundational dataset for all subsequent.

In determining the invasiveness of the alien insect species involved in this study, we adhered to the following four principles: (1) the species is not native to China; (2) it has been documented as invasive in China and has established a self-sustaining population; (3) it is listed in national invasive species catalogs or invasion atlases; and (4) its distribution and impacts in Guangxi have been recorded in both academic literature and local field surveys. Based on these principles, we primarily referred to the List of Invasive Alien Species in China (https://www.mee.gov.cn/gkml/hbb/bgg/201612/t20161226_373636.htm, accessed on 27 January 2026), the List of Key Managed Invasive Alien Species (https://fgs.moa.gov.cn/flfg/202211/t20221109_6415160.htm, accessed on 27 January 2026), Biological Invasions: Pictorial Handbook of Invasive Alien Animals in China [24], and the relevant scientific literature [25].

### 2.3. Statistical Analysis

The spatial patterns of IAI species richness were analyzed by defining the total number of recorded invasive alien insect species within each survey grid (10 km × 10 km) as the species richness for that grid. A natural breaks classification method was then used to categorize the grids into three richness levels: low richness (0–3 species), medium richness (4–7 species), and high richness (8–11 species), based on the species richness values obtained from all 84 survey grids.

We analyzed assemblage patterns and environmental drivers of IAI across three biodiversity priority areas using species occurrence data. Alpha diversity was evaluated by calculating species richness per grid, with differences among priority areas examined through the Kruskal–Wallis test. Beta diversity was partitioned into turnover (species replacement) and nestedness (species loss) components [26]. We conducted Principal Coordinates Analysis (PCoA) on Bray–Curtis dissimilarity matrices for visualization, and employed PERMANOVA to assess significant differences in assemblage structure among the priority areas [27].

To identify environmental drivers, we integrated 14 variables: six bioclimatic variables (mean annual temperature, bio1; maximum temperature of the warmest month, bio5; minimum temperature of the coldest month, bio6; annual precipitation, bio12; precipitation of the wettest month, bio13; and precipitation of the driest month, bio14) obtained from WorldClim (https://worldclim.org/, accessed on 17 September 2025) (30s resolution) and eight land cover types (cropland, forest, grassland, shrubland, wetland, water, artificial surface, and bare land) derived from GlobeLand30 V2020 (https://cloudcenter.tianditu.gov.cn/dataSource, accessed on 12 March 2026). We employed generalized linear mixed models (GLMMs) to investigate the effects of environmental factors on alpha diversity, while hierarchical partitioning analysis quantified the relative contributions of temperature, precipitation, and land cover variable groups to species richness [28,29]. For the beta diversity analysis, rare species (occurrence frequency < 10%) were excluded. We first conducted Detrended Correspondence Analysis (DCA); when the gradient length of the first axis exceeded 4 SD units, indicating a unimodal species response, we applied Canonical Correspondence Analysis (CCA) instead of linear models [30]. CCA, based on Hellinger-transformed species data, was utilized to evaluate the explanatory power of environmental factors, with significant variables (*p* < 0.05) selected. All statistical analyses were performed in R version 4.5.2 using packages such as vegan and mgcv.

## 3. Results

### 3.1. Species Composition of Invasive Alien Insect

A total of 19 species belonging to seven orders, 11 families, and 16 genera were recorded (Table 1). At the order level, Coleoptera (26.3%), Hymenoptera (21.1%), and Diptera (15.8%) were dominant. At the family level, Curculionidae (21.1%) was the most abundant family, followed by Tephritidae and Formicidae (15.8% each). At the genus level, *Bactrocera* (15.8%) was the dominant genus. The *Bactrocera cucurbitae* Bezzi, 1913 was the most frequently detected species (57.1% occurrence frequency), followed by the *Blattella germanica* Linnaeus, 1767 (53.6%), with the *Periplaneta americana* (Linnaeus, 1758), *Bemisia tabaci* (Gennadius, 1889), and *S*. *frugiperda* all occurring at 41.7% frequency. Most species (n = 11) had occurrence frequencies between 10% and 30%, while four species [*Leptocybe invasa* Fisher & La Salle, 2004, *Trialeurodes vaporariorum* (Westwood, 1856), *Sitophilus oryzae* Hustache, A., 1930, and *Bactrocera correcta* (Bezzi, 1916)] were rare, with frequencies below 5%.

### 3.2. Invasive Alien Insect Diversity

**Figure 2:**
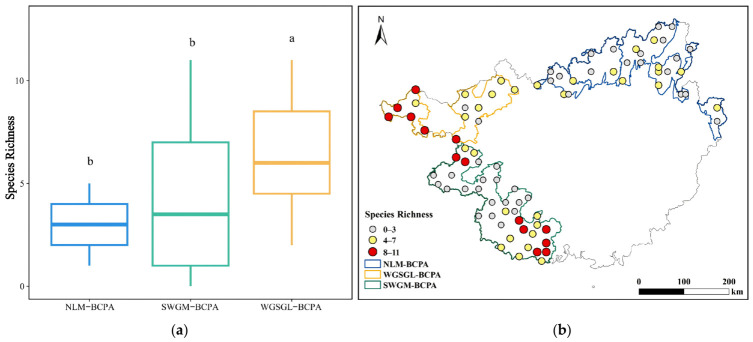
Spatial heterogeneity (**a**) and distribution (**b**) of IAI species richness in the Guangxi Biodiversity Conservation Priority Area (different lowercase letters in panel A indicate significant differences at *p* < 0.05).

The Kruskal–Wallis test indicated significant differences in species richness among the three priority areas (*p* < 0.05). Post hoc pairwise comparisons revealed that WGSGL-BCPA exhibited significantly higher species richness compared to SWGM-BCPA and NLM-BCPA, with no significant variance between SWGM-BCPA and NLM-BCPA (Figure 2a). The spatial distribution of species richness (Figure 2b) demonstrated that out of the 84 survey grids, the majority consisted of low-richness grids (0–3 species; *n* = 40) and medium-richness grids (4–7 species; *n* = 30), while high-richness grids (8–11 species; *n* = 14) were sparsely distributed. High-richness grids were predominantly concentrated in the southern region of SWGM-BCPA and the western area of WGSGL-BCPA. Remarkably, NLM-BCPA did not record any grids with high species richness (8–11 species), indicating distinctive regional characteristics.

**Figure 3:**
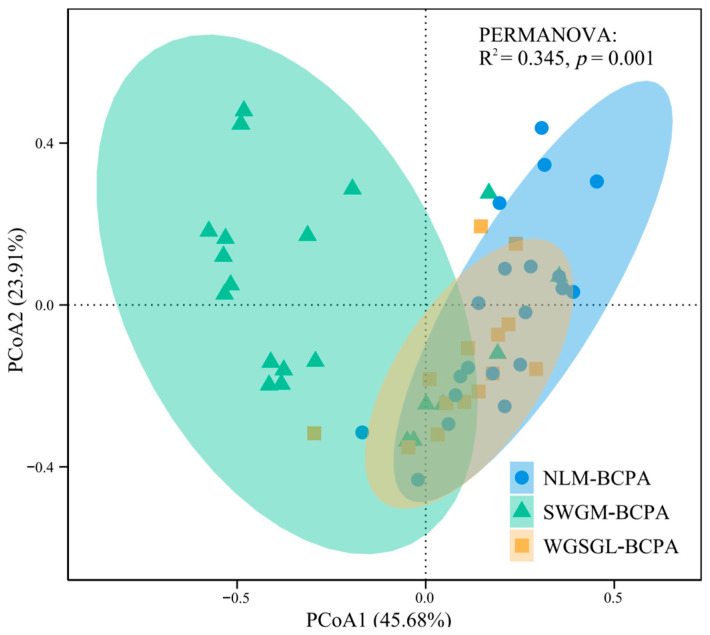
PCoA ordination visualizing β-diversity patterns of invasive insect assemblage based on Bray–Curtis dissimilarity.

Principal Coordinates Analysis (PCoA) utilizing the Bray–Curtis dissimilarity matrix indicated that PCoA1 accounted for 45.68% of the variation, while PCoA2 explained 23.91% (Figure 3). The three priority areas exhibited relatively distinct clusters within the ordination space, revealing significant differences in invasive insect assemblages’ composition across habitats (PERMANOVA, R^2^ = 0.345, and *p* = 0.001) (Figure 3). Beta diversity partitioning, based on the Sørensen dissimilarity index, yielded a total beta diversity of 0.645 ± 0.268. The turnover component contributed 0.474 ± 0.373, whereas the nestedness component was 0.171 ± 0.203, suggesting that species replacement, rather than nestedness, primarily drove assemblage differences.

### 3.3. Environmental Drivers of Invasive Alien Insect Diversity

**Figure 4:**
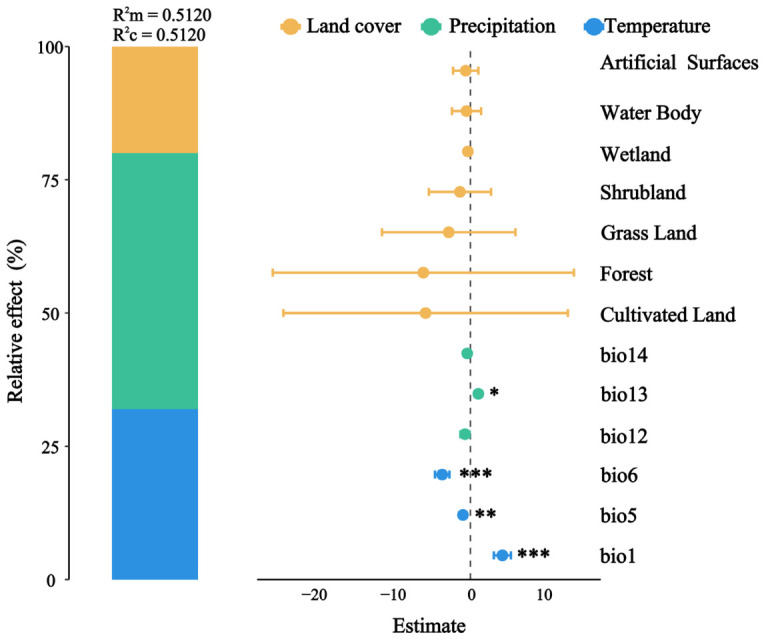
Hierarchical variance partitioning based on generalized linear mixed models (GLMMs) for effects of predictors on alien species richness (R^2^m: variance explained by fixed factors; R^2^c: variance explained by both fixed and random factors; the star indicates significance threshold: * *p* < 0.05, ** *p* < 0.01, *** *p* < 0.001).

Hierarchical partitioning analysis utilizing generalized linear mixed models (GLMMs) indicated that fixed effects, including temperature, precipitation, and land cover types, accounted for 51.2% of the variation in IAI species richness (marginal R^2^ = 0.512) (Figure 4). The conditional R^2^ of 0.512 suggested that random effects did not explain any additional variance, thereby highlighting environmental filtering as the predominant mechanism at the regional scale. The relative contributions of the three predictor groups were as follows: precipitation (48.0%) > temperature (32.0%) > land cover (20.0%), which positioned precipitation as the primary driver of IAI species richness (Figure 4). The GLMM results demonstrated a significant positive correlation between species richness and mean annual temperature (bio1, Est. = 1.406, SE = 0.321, and *p* < 0.001) as well as with precipitation during the wettest month (bio13, Est. = 1.066, SE = 0.458, and *p* < 0.05). Conversely, species richness exhibited a negative correlation with the maximum temperature of the warmest month (bio5, Est. = −0.993, SE = 0.342, and *p* < 0.01) and the minimum temperature of the coldest month (bio6, Est. = −3.442, SE = 0.876, and *p* < 0.001).

**Figure 5:**
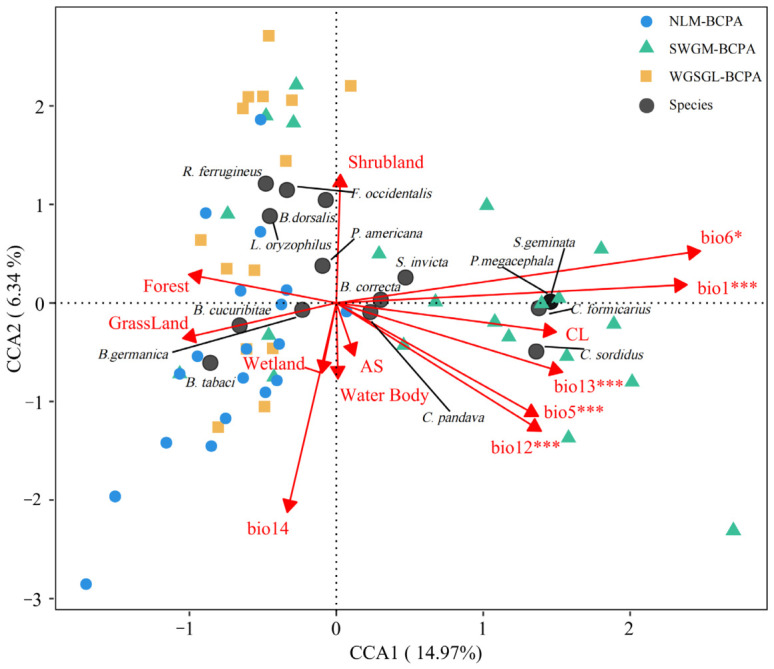
Canonical Correspondence Analysis (CCA) ordination of invasive insect assemblages composition based on Hellinger distance-transformed data (arrows indicate environmental drivers: bio1 = annual mean temperature; CL = cultivated land; and AS = artificial surfaces). The star indicates significance threshold: * *p* < 0.05, *** *p* < 0.001).

Canonical Correspondence Analysis (CCA) was employed to investigate the factors influencing beta diversity variation. The CCA model proved significant, as indicated by the Monte Carlo permutation test (999 permutations, F = 3.374, and *p* = 0.001). The first two axes, CCA1 and CCA2, accounted for 14.97% and 6.34% of the species–environment variation, respectively (Figure 5). Only climatic variables (bio1, bio5, bio6, bio12, and bio13) achieved statistical significance (*p* < 0.05), whereas indicators of human activity (cropland and artificial surface) did not show significance. In the ordination space, assemblages from NLM-BCPA were primarily located in areas characterized by lower temperature gradients (low bio1 and bio5), potentially reflecting assemblages of cold-adapted invasive species. Conversely, assemblages from SWGM-BCPA clustered in warm and humid regions (high bio1, bio12, and bio13), demonstrating a distinct preference for warm and moist environments. Assemblages from WGSGL-BCPA exhibited a broader distribution across various climate gradients, suggesting greater environmental tolerance. At the species level, tropical invasive species that favor warm conditions (e.g., *S. invicta*; *C. sordidus*) were associated with elevated bio1 and bio5, while species adapted to seasonal cold (e.g., *F. occidentalis*) were found in the opposite direction.

## 4. Discussion

### 4.1. Compositional Characteristics of Invasive Alien Insects

Our investigation unveiled that the invasive insect assemblage in the study area was predominantly comprising Coleoptera, Hymenoptera, and Diptera, with *B. cucurbitae* emerging as the most commonly identified species. This composition, characterized by the prevalence of Coleoptera (especially the family Curculionidae), mirrors worldwide trends in insect invasions, highlighting the substantial biosecurity threats linked to the international trade of agricultural and forestry goods [3,31]. Moreover, the notable occurrence rate of the *Bactrocera* genus, exemplified by *B. cucurbitae*, provides direct evidence that the global fruit trade serves as a primary conduit for disseminating such highly adaptable, polyphagous pests [32,33]. These results collectively suggest that the establishment of the local assemblages are significantly influenced by a common species pool shaped by global trade dynamics and subsequently sieved through local environmental mechanisms [6]. The distinctive contribution of this study resides in its innovative spatial perspective. In contrast to most prior research that has concentrated on regions of intense human activity, such as ports, farmlands, and urban centers, this investigation was systematically conducted within Biodiversity Conservation Priority Areas. As a result, the number of species recorded (19 species) was significantly lower than the provincial-level baseline data, which indicates 107 species known for Guangxi [34]. This discrepancy does not signify a lack of data; rather, it reflects the relatively low levels of direct human disturbance within the protected area. This finding demonstrates that even in regions designated for the conservation of native ecosystems, the infiltration and establishment of alien insects have already occurred. However, the assemblage structure, characterized by a few pioneer species with exceptionally strong dispersal and adaptive capabilities, and the invasion pressure differ from those observed in highly anthropogenically disturbed habitats. This discovery aligns with the regional macro-scale pattern, revealing a gradient in IAI numbers across southern China, with high values recorded in Yunnan (121 species), Guangdong (112 species), and Guangxi (107 species). These findings collectively suggest that international trade and climatic suitability are core drivers of this phenomenon. Furthermore, they underscore that within protected areas, the species’ inherent climatic adaptability, niche breadth, and phenotypic plasticity serve as critical filters for successful establishment [16,35].

Management practices necessitate differentiated and synergistic strategies. First, it is essential to enhance attention and fortify the biosecurity barrier function of Biodiversity Conservation Priority Areas. Specialized monitoring and quarantine interception belts should be established around these areas and at key entry points to target high-risk taxa associated with trade, such as fruit flies and weevils [6,36]. Second, habitat-specific management priorities must be implemented: in regions of intense human activity, the primary focus should be on reducing propagule pressure; conversely, in conservation priority areas, the strategy should prioritize the early detection and rapid eradication of established pioneer species, and at initiatives aimed at restoring the inherent resilience of ecosystems to combat invasive species capable of crossing natural barriers.

### 4.2. Diversity Patterns and Key Environmental Drivers

Our analyses revealed significant differences in species richness among the priority areas, with assemblage dissimilarities primarily driven by species turnover (beta-diversity). Climatic factors emerged as the primary determinants influencing both alpha and beta diversity within these invasive insect assemblages. Specifically, climate variables, including precipitation and temperature, collectively accounted for the majority of the variation in species richness (Figure 4). This finding supports a fundamental principle in invasion ecology: climate suitability serves as a crucial abiotic filter that determines the successful establishment and population growth of invasive species [17,37]. While numerous studies highlight the critical role of human-mediated factors, such as trade and propagule pressure during the transport and introduction stages [6,21], our results indicate that once introduction occurs, the establishment and local abundance of IAI are primarily constrained by climatic conditions. This observation aligns with global analyses that identify climate matching as a vital predictor of invasion success [38].

IAI species richness exhibited a positive correlation with annual mean temperature (bio1) and precipitation during the wettest month (bio13), while it showed a negative correlation with the maximum temperature of the warmest month (bio5) and the minimum temperature of the coldest month (bio6). This pattern indicates that warmer average conditions and sufficient moisture during the growing season create favorable baseline conditions [36,39], In contrast, temperature extremes impose physiological constraints and distribution limits [40,41]. These mechanisms collectively elucidate the observed spatial patterns in species richness (Figure 2). For example, the area exhibiting the highest richness (e.g., WGSGL-BCPA), characterized by shrubland habitats, may benefit from relatively humid and stable microclimates that mitigate macroclimatic extremes (Figure 5) [42], Additionally, greater plant diversity in shrublands may provide more host resources for phytophagous insects [43]. Conversely, thermal stress resulting from high (e.g., SWGM-BCPA) or low (e.g., NLM-BCPA) temperatures may directly or indirectly restrict species survival, leading to reduced richness in these regions (Figure 5).

In contrast, local land use variables, such as cropland and artificial surface area, exhibited relatively limited explanatory power for diversity patterns at the regional (10 km grid) scale. This limitation can be attributed to several interrelated mechanisms. First, climate serves as a large-scale environmental filter, establishing fundamental physiological tolerance boundaries. These continuous gradients directly drive the spatial replacement of species composition, resulting in high turnover, a pattern corroborated by our beta-diversity partitioning result [6,36]. Second, while land use change often facilitates invasion by creating disturbed, resource-rich environments [44], the study areas are designated as protected priority zones. In comparison to intensively modified agricultural or urban landscapes, the intensity gradient of human modification in this context is relatively weaker or patchier, and its independent statistical signal may be obscured by the strong, continuous climatic gradient. Third, the impact of human activity may be more pronounced during the introduction phase due to propagule pressure, whereas post-introduction establishment and persistence are more critically dependent on climatic suitability [6,12].

### 4.3. Limitations and Future Directions

This study offers significant insights; however, several limitations must be acknowledged. Data derived from a single survey period may not adequately capture the temporal dynamics of assemblages, including seasonal variations or long-term establishment trends. Our active sampling methods (such as sweeping nets, manual collection, and light trapping) may underestimate invasive species that are hidden, ground dwelling, or nocturnal, which can be better captured through passive traps (such as traps, pheromones, or window traps). Additionally, while we quantified essential climate and land cover variables, other critical drivers highlighted in the literature were not directly measured. These include propagule pressure linked to specific trade pathways and commodity types [6,21,45], which could further elucidate the differences in initial species pools across various areas. The impacts of biotic interactions, such as competition with native species or mutualism with other invaders [17,46] were also not assessed.

Future research could enhance this study by integrating long-term monitoring data to confirm the stability of observed assemblage patterns and evaluate invasion trajectories. To achieve a more complete inventory of invasive insect assemblages, future surveys should employ a combination of passive traps (pitfall traps, window traps, and bait traps) alongside active methods. By merging field assemblage data with dispersal pathway analysis, such as associating specific invaders with nearby trade ports or agricultural hubs, researchers could elucidate the roles of environmental suitability and human-mediated dispersal in shaping invasion patterns. Furthermore, applying the assemblage-level findings from this research to species distribution models could enhance regional-scale predictions. Recent studies have underscored the importance of integrated modeling approaches that integrate fine-scale microclimate data and species interactions to improve the precision of invasion risk forecasts [42,47]. This is crucial for guiding proactive biosecurity and conservation management strategies in the context of global change.

## 5. Conclusions

This study systematically assessed the assembly of invasive insect assemblages within three terrestrial Biodiversity Conservation Priority Areas (BCPAs) in Guangxi, China. A total of 19 IAI species were identified. The key finding reveals that the assembly of these assemblages is predominantly governed by climatic filtering. Although international trade and other human activities serve as the initial sources and drivers of species introductions, the successful establishment and subsequent assembly of IAI are primarily determined by local climatic suitability. Specifically, precipitation and temperature function as the key environmental filters, significantly shaping spatial patterns of species richness and assemblage composition dissimilarity, and their effects substantially outweigh those of local land use types. High beta diversity, largely attributable to species turnover, further supports the role of climatic gradients in filtering species distributions. These findings indicate that even within BCPAs, climate conditions play a decisive role in the colonization patterns of IAI. Therefore, future biosecurity management and conservation strategies in BCPAs should not only reduce anthropogenic propagule pressure but also incorporate climatic suitability as a core criterion for invasion risk assessment and monitoring prioritization, guided by fine-scale climate-matching models and field assemblage survey data.

## Figures and Tables

**Table 1:** List of invasive alien insect, their occurrence frequency and distribution in the Guangxi Biodiversity Conservation Priority Area (occurrence frequency is defined as the ratio of grids where the species occurred to the total surveyed grids; “+” indicates that the species appears in the area).

Order	Family	Genus	Species	Occurrence Frequency (%)	NLM-BCPA	SWGM-BCPA	WGSGL-BCPA
Blattodea	Phyllodromiidae	*Blattella*	*Blattella germanica* Linnaeus, 1767	53.6	+	+	+
	Blattidae	*Periplaneta*	*Periplaneta americana* (Linnaeus, 1758)	41.7	+	+	+
Homoptera	Aleyrodidae	*Bemisia*	*Bemisia tabaci* (Gennadius, 1889)	41.7	+	+	+
		*Trialeurodes*	*Trialeurodes vaporariorum* (Westwood, 1856)	2.4	+		
Coleoptera	Curculionidae	*Lissorhoptrus*	*Lissorhoptrus oryzophilus* Kuschel, 1952	13.1	+	+	+
		*Rhynchophorus*	*Rhynchophorus ferrugineus* (Olivier, A.G., 1791)	14.3		+	+
		*Cosmopolites*	*Cosmopolites sordidus* (Germar, E.F., 1823)	26.2	+		+
		*Sitophilus*	*Sitophilus oryzae* Hustache, A., 1930	1.2	+		
	Cyladidae	*Cylas*	*Cylas formicarius* (Fabricius, 1798)	15.5		+	
Diptera	Tephritidae	*Bactrocera*	*Bactrocera cucuribitae* Bezzi, 1913	57.1	+	+	+
			*Bactrocera dorsalis* (Hendel, 1912)	14.3		+	+
			*Bactrocera correcta* (Bezzi, 1916)	1.2		+	
Lepidoptera	Noctuidae	*Spodoptera*	*Spodoptera frugiperda* Smith, J.E., 1797	41.7	+	+	+
	Lycaenidae	*Lycaenidae*	*Chilades pandava* (Horstfield, 1829)	28.6	+	+	+
Hymenoptera	Eulophidae	*Leptocybe*	*Leptocybe invasa* Fisher & La Salle, 2004	3.6		+	+
	Formicidae	*Solenopsis*	*Solenopsis invicta* Buren, 1972	26.2	+	+	+
			*Solenopsis geminata* (Fabricius, 1804)	10.7		+	
		*Pheidole*	*Pheidole megacephala* (Fabricius, 1793)	10.7		+	
Thysanoptera	Thripidae	*Frankliniella*	*Frankliniella occidentalis* (Pergande, 1895)	10.7		+	+

## Data Availability

The datasets generated during and/or analyzed during the current study are available from the corresponding author on reasonable request.
